# Supporting safe and gradual reduction of long‐term benzodiazepine receptor agonist use: Development of the SAFEGUARDING‐BZRAs toolkit using a codesign approach

**DOI:** 10.1111/hex.13547

**Published:** 2022-06-07

**Authors:** Tom Lynch, Cristín Ryan, Colin Bradley, D. Foster, Christy Huff, Sharon Hutchinson, Nicole Lamberson, Lily Lynch, Cathal Cadogan

**Affiliations:** ^1^ School of Pharmacy and Biomolecular Sciences Royal College of Surgeons in Ireland Dublin Ireland; ^2^ School of Pharmacy and Pharmaceutical Sciences Trinity College Dublin Dublin Ireland; ^3^ Department of General Practice University College Cork Cork Ireland; ^4^ Benzodiazepine Action Work Group Colorado Consortium for Prescription Drug Abuse Prevention Aurora Colorado USA; ^5^ Benzodiazepine Information Coalition Midvale Utah USA; ^6^ Dublin Ireland; ^7^ Virginia USA; ^8^ Belgrade Serbia

**Keywords:** behaviour, benzodiazepines, codesign, discontinuation, Theoretical Domains Framework, toolkit, Z‐drugs

## Abstract

**Introduction:**

Long‐term benzodiazepine receptor agonist (BZRA) use persists in healthcare settings worldwide and poses risks of patient harm.

**Objective:**

This study aimed to develop an intervention to support discontinuation of long‐term BZRA use among willing individuals.

**Methods:**

The intervention development process aligned with the UK Medical Research Council's complex intervention framework. This involved a previous systematic review of brief interventions targeting long‐term BZRA use in primary care and qualitative interviews based on the Theoretical Domains Framework that explored barriers and facilitators to discontinuing long‐term BZRA use. A codesign approach was used involving an active partnership between experts by experience, researchers and clinicians. Intervention content was specified in terms of behaviour change techniques (BCTs).

**Results:**

The SAFEGUARDING‐BZRAs (Supporting sAFE and GradUAl ReDuctIon of loNG‐term BenZodiazepine Receptor Agonist uSe) toolkit comprises 24 BCTs and includes recommendations targeted at primary care‐based clinicians for operationalizing each BCT to support individuals with BZRA discontinuation.

**Conclusion:**

The SAFEGUARDING‐BZRAs toolkit has been developed using a systematic and theory‐based approach that addresses identified limitations of previous research. Further research is needed to assess its usability and acceptability by service users and clinicians, as well as its potential to effectively support safe and gradual reduction of long‐term BZRA use.

**Patient or Public Contribution:**

The qualitative interview phase included patients as participants. The codesign process included ‘experts by experience’ with either current or previous experience of long‐term BZRA use as collaborators.

## 1 INTRODUCTION

Long‐term use of benzodiazepine receptor agonists (BZRAs) is a persistent clinical challenge in healthcare settings worldwide, despite guidelines, since the 1980s, repeatedly recommending short‐term use (≤4 weeks).^1^ Many countries have reported limited or no significant change in BZRA prescribing levels in recent years.^2^, ^3^, ^4^ A longitudinal analysis of prescriptions issued on the main public health scheme in Ireland from 2005 to 2015 reported a significant overall reduction in benzodiazepine prescribing, which was offset by significant increases in the prescribing of Z‐drugs (e.g., zopiclone).^5^ This is consistent with trends observed in other countries.^4^, ^6^ There is no consensus regarding the duration that constitutes long‐term BZRA use. A previous systematic review of register‐based studies on long‐term BZRA use reported that the most commonly used definition was 6 months or longer and identified a prevalence of approximately 3% in the general population, with the highest prevalence consistently found in older adults (≥65 years).^7^ Age‐related physiological changes make older adults more susceptible to BZRA‐related adverse effects^8^ such as falls and subsequent fractures, as well as road traffic accidents.^9^

To address the issue of long‐term BZRA use, various interventions have been evaluated including brief intervention‐based approaches (e.g., short consultations with healthcare professionals recommending BZRA discontinuation), cognitive behavioural therapy and pharmacological treatments (e.g., antidepressants, anticonvulsants).^10^, ^11^, ^12^, ^13^ However, translating effective interventions into clinical practice remains problematic due to deficits in their development and reporting. Furthermore, previous studies have tended to focus on reducing prescribing of benzodiazepines by prescribers rather than on how to support service users discontinue their use of benzodiazepines.^14^ A recent systematic review showed that brief interventions delivered by healthcare professionals in primary care are effective in helping patients to reduce and discontinue long‐term BZRA use.^12^ However, the review also highlighted that previous interventions often lacked an underpinning theory base, were poorly described and did not indicate how or if there was any service user buy‐in.^12^ This poses challenges in terms of understanding the mechanisms by which the interventions worked, as well as replicating effective interventions in clinical practice.

A multiphase research project has been undertaken to develop a theory‐based intervention to support discontinuation of long‐term BZRA use in primary care. The project is being conducted in accordance with the UK Medical Research Council's framework for developing and evaluating complex interventions.^15^ The first phase of the project involved establishing the current evidence base by systematically reviewing the evidence for brief interventions targeting long‐term BZRA use in primary care.^12^ The second phase focused on incorporating an appropriate theory base into the intervention's development by using the Theoretical Domains Framework (TDF) to explore behavioural determinants of discontinuing long‐term BZRA use from a service user perspective.^16^ The TDF is an integrated framework of 33 behaviour change theories. It comprises domains that are considered mediators (i.e., barriers, facilitators) of behaviour change. The first version of the TDF (TDFv1) comprises 12 theoretical domains^17^ and the second version (TDFv2) consists of 14 domains.^18^ Both versions of the TDF are widely used, and given the similarity between them, either can be used depending on researchers' familiarity and preference.^19^

**Figure 1:**
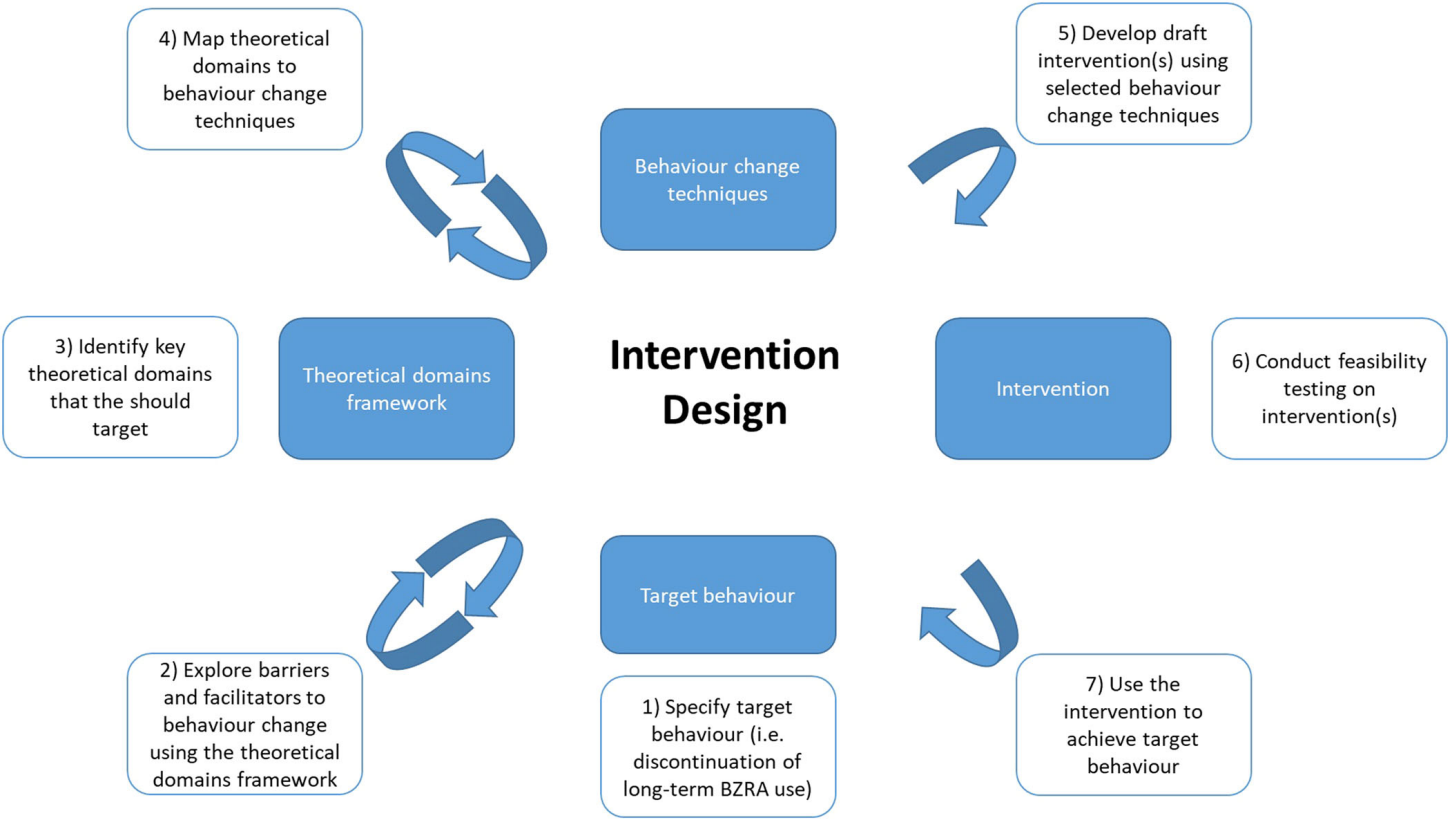
Systematic process of intervention development using the Theoretical Domains Framework (adapted from Cadogan et al).^22^ BZRA, benzodiazepine receptor agonist.

Domains that need to be targeted to achieve changes in the target behaviour can then be mapped to behaviour change techniques (BCTs), which form the intervention's active components (Figure 1).^20^ BCTs are defined as observable, replicable and irreducible components of an intervention, which can be used to change behaviour.^21^ The BCT Taxonomy (v1) consists of 93 BCTs and provides a method for specifying and reporting the content of behaviour change interventions.^21^

The aim of this study was to develop an intervention to support discontinuation of long‐term use of BZRAs using a codesign approach. The objectives were to:

1. Map theoretical domains to BCTs;
2. Select BCTs to include in the intervention; and
3. Operationalize selected BCTs as part of the intervention.

## 2 METHODS

The intervention development process followed a systematic approach comprising several steps as illustrated in Figure 1 and outlined below. This process was based on previous TDF‐based intervention development studies.^22^, ^23^ and incorporated a codesign approach, whereby lay individuals and professionals worked as equals during the research process.^24^ The codesign approach was guided by previous research and followed established principles of authentic participation and collaboration.^25^ The codesign team consisted of the lead researcher (pharmacist undertaking a PhD) and two research supervisors (academic pharmacists with extensive experience in the development of behaviour change interventions), an academic general practitioner and five ‘experts by experience’ with either current or previous experience of long‐term BZRA use. Team members were based in Europe (*n* = 5) and the United States (*n* = 3).

The initial qualitative component of the intervention development process is reported in a separate publication^16^ and summarized briefly below. This paper focuses on the process of mapping theoretical domains to BCTs and selecting and operationalizing BCTs as part of an intervention. Ethical approval was granted by the RCSI Research Ethics Committee (REC reference: 1727).

### 2.1 Initial steps of the intervention development process (Steps 1–3)

Discontinuation of long‐term BZRA use by patients was the target behaviour that this study focused on. As part of the previous qualitative study, semi‐structured interviews were conducted with two cohorts of participants comprising individuals based in the Republic of Ireland with either current (*n* = 15) or previous experience (*n* = 13) of long‐term BZRA use. For the purpose of this study, long‐term BZRA use was defined based on ≥3 months' supply in the previous year. The interviews explored participants' perceptions of barriers and facilitators to discontinuing long‐term use of BZRAs using a TDF‐based topic guide. The interviews were audio‐recorded, transcribed and analysed using the framework method.^26^

This study highlighted that, while commonalities existed between participants in terms of perceived barriers and facilitators to discontinuing BZRA use, individual participants had different experiences of the identified determinants of BZRA discontinuation. This prevented a meaningful selection of key domains as the relevance of individual domains varied according to individual participants. Therefore, all domains were considered potentially relevant, with the need for any future intervention to be tailored according to individuals' specific needs. A detailed outline of the interview findings is presented in a related publication.^16^

### 2.2 BCT mapping and selection (Steps 3 and 4)

The BCT mapping process focused on identifying BCTs to target theoretical domains as part of the intervention. This process was conducted using an established mapping matrix.^27^ This matrix comprised a table of BCTs that have been reliably allocated to the TDF (version 2) domains by a panel of behaviour change experts.^27^ This was supplemented by a secondary mapping reference source^20^ to account for two domains that had not been mapped to BCTs within the previous matrix (i.e., ‘Social/professional role and identity’, ‘Memory attention and decision processes’). Three members of the research team (T. L., C. C., C. R.) independently reviewed all of the BCTs that had been mapped to TDF domains in each reference source and documented their decisions (include, exclude, unsure) as to whether each BCT should be included in a long list for discussion with the other members of the codesign team. A consensus‐based approach was used to reach final decisions. This selection process focused on removing BCTs that were clearly irrelevant or unsuitable due to practical considerations. For example, BCT 2.6 Biofeedback (‘Provide feedback about the body [e.g., physiological or biochemical state] using an external monitoring device as part of a behaviour change strategy’) was not relevant to this study as there are no suitable or available devices to provide this type of feedback in relation to BZRA discontinuation. All reasons for excluding BCTs were documented. The remaining BCTs were then reviewed and discussed regarding inclusion in the intervention at the codesign team meetings.

Two codesign team meetings were held online in July 2021. Both meetings were chaired by the lead researcher (T. L.). During the first meeting, the researcher presented a summary of findings from previous work packages (systematic review, qualitative interviews) and explained the proposed approach to the intervention's development. This provided a means of orientating the team members to the project and addressing any queries. The long list of BCTs was presented and discussed, and each member of the codesign team was asked to independently document his or her decision (include, exclude, unsure) as to whether each BCT should be included in a short list for potential inclusion in the final intervention. This process was conducted using Alchemer® survey software and team members had the option of adding free‐text comments to support their decisions. The a priori decision rule was that 70% of team members had to be in agreement regarding the inclusion/exclusion of a BCT.^28^ All other BCTs were then discussed at a follow‐up meeting (below).

### 2.3 Developing draft intervention(s) using selected BCTs (Step 5)

During the second codesign team meeting, findings from the previous BCT review exercise were presented and discussed with a particular focus on BCTs for which there was no clear decision regarding inclusion/exclusion. A finalized list of BCTs for inclusion in the intervention was agreed using a consensus‐based approach. The same a priori decision rule was used again (70% of the team members had to be in agreement regarding inclusion/exclusion of a BCT).

### 2.4 Selecting draft intervention(s) for further testing (Step 6)

Possible ways in which the BCTs could be operationalized as part of the intervention were then discussed. The team looked to develop draft interventions with specified modes of BCT delivery. In the event that multiple draft interventions were proposed, it was intended that team members would independently assess each of the draft interventions using the APEASE (Affordability, Practicability, Effectiveness/cost‐effectiveness, Acceptability, Side‐effects/safety, Equity) criteria through online polling software. The APEASE criteria are intended to assist researchers in developing and evaluating interventions.^29^ In applying the criteria, team members would be asked to assess each criterion in terms of each draft intervention's strengths and limitations. These assessments would be collated and then reviewed and discussed by the team. The same consensus‐based approach was to be used for final decisions regarding the selection of one draft intervention for further testing.

## 3 RESULTS

### 3.1 BCT mapping and selection (Steps 3 and 4)

In total, 60 BCTs were mapped to individual TDF domains. Following independent review by the three team members, 31 BCTs were considered irrelevant to the target behaviour or unsuitable due to practical considerations. The reasons for exclusion of BCTs are documented in Appendix S1. The remaining 29 BCTs formed the long list that was presented at the first codesign meeting (Table 1). All 14 domains had linked BCTs included in the long list.

**Table 1:** Long list of behaviour change techniques reviewed by the codesign team for potential inclusion in the intervention.

Theoretical domain	Behaviour change technique	Definition	Results of first‐round voting (>70% required for inclusion)	Results of second‐round voting (>70% required for inclusion)
Knowledge	Information about health consequences (BCT 5.1)	Provide information (e.g., written, verbal, visual) about health consequences of performing the behaviour	Include: 100%	N/A
			Exclude: 0%	
			Unsure: 0%	
	Feedback on behaviour (BCT 2.2)	Monitor and provide informative or evaluative feedback on the performance of the behaviour (e.g., form, frequency, duration, intensity)	Include: 100%	N/A
			Exclude: 0%	
			Unsure: 0%	
	Distraction (BCT 12.4)	Advise or arrange to use an alternative focus for attention to avoid triggers for unwanted behaviour cues for the behaviour, including changing daily or weekly routines	Include: 100%	N/A
			Exclude: 0%	
			Unsure: 0%	
Skills	Graded task (BCT 8.7)	Set easy‐to‐perform tasks, making them increasingly difficult, but achievable, until behaviour is performed	Include: 92.3%	N/A
			Exclude: 7.7%	
			Unsure: 0%	
	Habit reversal (BCT 8.4)	Prompt rehearsal and repetition of an alternative behaviour to replace an unwanted habitual behaviour	Include: 61.5%	Include: 100%
			Exclude: 7.7%	Exclude: 0%
			Unsure: 30.8%	Unsure: 0%
	Body changes (BCT 12.6)	Alter body structure, functioning or support directly to facilitate behaviour change	Include: 92.3%	N/A
			Exclude: 7.7%	
			Unsure: 0%	
Social/professional role and identity	Social comparison (BCT 6.2) [also included under the ‘social influences’ domain]	Draw attention to others' performance to allow comparison with the person's own performance Note: Being in a group setting does not necessarily mean that social comparison is actually taking place	Include: 11.1%	N/A
			Exclude: 77.8%	
			Unsure: 11.1%	
	Social support (emotional) (BCT 3.3) [also included under the ‘social influences’ domain]	Advise on, arrange or provide emotional social support (e.g., from friends, relatives, colleagues, ‘buddies’ or staff) for the performance of the behaviour	Include: 100%	N/A
			Exclude: 0%	
			Unsure: 0%	
	Social support (practical) (BCT 3.2) [also included under the ‘social influences’ domain]	Advise on, arrange or provide practical help (e.g., from friends, relatives, colleagues, ‘buddies’ or staff) for performance of the behaviour	Include: 100%	N/A
			Exclude: 0%	
			Unsure: 0%	
Beliefs about capabilities	Verbal persuasion to boost self‐efficacy (BCT 15.1) [also included under ‘optimism’ domain]	Tell the person that they can successfully perform the wanted behaviour, arguing against self‐doubts and asserting that they can and will succeed	Include: 100%	N/A
			Exclude: 0%	
			Unsure: 0%	
	Focus on past success (BCT 15.3)	Advise to think about or list previous successes in performing the behaviour (or parts of it)	Include: 100%	N/A
			Exclude: 0%	
			Unsure: 0%	
Optimism	Verbal persuasion to boost self‐efficacy (BCT 15.1) [also included under ‘beliefs about capabilities’ domain]	Tell the person that they can successfully perform the wanted behaviour, arguing against self‐doubts and asserting that they can and will succeed	Include: 100%	N/A
			Exclude: 0%	
			Unsure: 0%	
Beliefs about consequences	Salience of consequences (BCT 5.2)	Use methods specifically designed to emphasize the consequences of performing the behaviour with the aim of making them more memorable (goes beyond informing about consequences)	Include: 63.6%	N/A
			Exclude: 9.1%	
			Unsure: 27.3%	
	Comparative imagining of future outcomes (BCT 9.3)	Prompt or advise the imagining and comparing of future outcomes of changed versus unchanged behaviour	Include 90.9%	N/A
			Exclude: 9.1%	
			Unsure: 0%	
	Pros and cons (BCT 9.2)	Advise the person to identify and compare reasons for wanting (pros) and not wanting to (cons) change the behaviour (includes ‘Decisional balance’)	Include: 100%	N/A
			Exclude: 0%	
			Unsure: 0%	
Reinforcement	Social reward (BCT 10.4)	Arrange verbal or nonverbal reward if and only if there has been effort and/or progress in performing the behaviour (includes ‘Positive reinforcement’)	Include: 72.7%	N/A
			Exclude: 9.1%	
			Unsure: 18.2%	
	Self‐reward (BCT 15.4)	Prompt self‐praise or self‐reward if and only if there has been effort and/or progress in performing the behaviour	Include: 100%	N/A
			Exclude: 0%	
			Unsure: 0%	
Intentions	Commitment (BCT 1.9)	Ask the person to affirm or reaffirm statements indicating commitment to change the behaviour	Include: 30%	N/A
			Exclude: 40%	
			Unsure: 30%	
Goals	Goal setting (behaviour) (BCT 1.1)	Set or agree on a goal defined in terms of the behaviour to be achieved	Include: 90%	N/A
			Exclude: 0%	
			Unsure: 10%	
	Goal setting (outcome) (BCT 1.3)	Set or agree on a goal defined in terms of a positive outcome of wanted behaviour	Include: 50%	N/A
			Exclude: 10%	
			Unsure: 40%	
	Review outcome goal(s) (BCT 1.7)	Review outcome goal(s) jointly with the person and consider modifying goal(s) in light of achievement. This may lead to resetting the same goal, a small change in that goal or setting a new goal instead of or in addition to the first	Include: 70%	N/A
			Exclude: 10%	
			Unsure: 20%	
	Review behaviour goal(s) (BCT 1.5)	Review behaviour goal(s) jointly with the person and consider modifying goal(s) or behaviour change strategy in light of achievement. This may lead to re‐setting the same goal, a small change in that goal or setting a new goal instead of (or in addition to) the first or no change	Include: 100%	N/A
			Exclude: 0%	
			Unsure: 0%	
	Action planning (BCT 1.4) [also included under ‘memory, attention and decision processes’ domain]	Prompt detailed planning of performance of the behaviour (must include at least one of context, frequency, duration and intensity). Context may be environmental (physical or social) or internal (physical, emotional or cognitive) (includes ‘Implementation Intentions’)	Include: 66.7%	N/A
			Exclude: 11.1%	
			Unsure: 22.2%	
Memory, attention and decision processes	Action planning (BCT 1.4) [also included under the ‘goals’ domain]	Prompt detailed planning of performance of the behaviour (must include at least one of context, frequency, duration and intensity). Context may be environmental (physical or social) or internal (physical, emotional or cognitive) (includes ‘Implementation Intentions’)	Include: 66.7%	N/A
			Exclude: 11.1%	
			Unsure: 22.2%	
	Prompts/cues (BCT 7.1) [also included under the ‘environmental context and resources’ domain]	Introduce or define environmental or social stimulus with the purpose of prompting or cueing the behaviour. The prompt or cue would normally occur at the time or place of performance	Include: 88.9%	N/A
			Exclude: 0%	
			Unsure: 11.1%	
	Self‐monitoring of behaviour (BCT 2.3) [also included under the ‘behavioural regulation’ domain]	Establish a method for the person to monitor and record their behaviour(s) as part of a behaviour change strategy	Include: 100%	N/A
			Exclude: 0%	
			Unsure: 0%	
Environmental context and resources	Adding objects to the environment (BCT 12.5)	Add objects to the environment to facilitate performance of the behaviour	Include: 92.3%	N/A
			Exclude: 7.7%	
			Unsure: 0%	
	Avoidance/reducing exposure to cues for the behaviour (BCT 12.3)	Advise on how to avoid exposure to specific social and contextual/physical	Include: 46.2%	N/A
			Exclude: 30.8%	
			Unsure: 23.1%	
	Prompts/cues (BCT 7.1) [also included under the ‘memory, attention and decision’ processes]	Introduce or define environmental or social stimulus with the purpose of prompting or cueing the behaviour. The prompt or cue would normally occur at the time or place of performance	Include: 88.9%	N/A
			Exclude: 0%	
			Unsure: 11.1%	
Social influences	Social comparison (BCT 6.2) [also included under the ‘social/professional role and identity’ domain]	Draw attention to others' performance to allow comparison with the person's own performance Note: being in a group setting does not necessarily mean that social comparison is actually taking place	Include: 11.1%	N/A
			Exclude: 77.8%	
			Unsure: 11.1%	
	Social support (emotional) (BCT 3.3) [also included under the ‘social/professional role and identity’ domain]	Advise on, arrange or provide emotional social support (e.g., from friends, relatives, colleagues, ‘buddies’ or staff) for the performance of the behaviour	Include: 100%	N/A
			Exclude: 0%	
			Unsure: 0%	
	Social support (practical) (BCT 3.2) [also included under the ‘social/professional role and identity’ domain]	Advise on, arrange or provide practical help (e.g., from friends, relatives, colleagues, ‘buddies’ or staff) for performance of the behaviour	Include: 100%	N/A
			Exclude: 0%	
			Unsure: 0%	
Emotion	Reduce negative emotions (BCT 11.2)	Advise on ways of reducing negative emotions to facilitate performance of the behaviour (includes ‘Stress Management’)	Include: 88.8%	N/A
			Exclude: 11.1%	
			Unsure: 0%	
	Monitoring of emotional consequences (BCT 5.4)	Prompt assessment of feelings after attempts at performing the behaviour	Include: 88.9%	N/A
			Exclude: 0%	
			Unsure: 11.1%	
	Information about emotional consequences (BCT 5.1)	Provide information (e.g., written, verbal, visual) about emotional consequences of performing the behaviour	Include: 100%	N/A
			Exclude: 0%	
			Unsure: 0%	
Behavioural regulation	Self‐monitoring of behaviour (BCT 2.3) [also included under the ‘memory, attention and decision making processes’ domain]	Establish a method for the person to monitor and record their behaviour(s) as part of a behaviour change strategy	Include: 100%	N/A
			Exclude: 0%	
			Unsure: 0%	
Not applicable	Credible Source (BCT 9.1)	Present verbal or visual communication from a credible source in favour of or against the behaviour	N/A [this BCT was identified following discussion at the first codesign meeting]	Include: 100%
	[BCT has not been mapped to a theoretical domain]			
				Exclude: 0%
				Unsure: 0%

Abbreviation: BCT, behaviour change technique.

Following review of the long list of BCTs and discussion, the codesign team voted to include 23 BCTs, which were discussed further at the second codesign team meeting: ‘Goal setting (behaviour)’ (BCT 1.3), ‘Review behaviour goal(s)’ (BCT 1.5), ‘Review outcome goal(s)’ (BCT 1.7), ‘Feedback on behaviour’ (BCT 2.2), ‘Self‐monitoring of behaviour’ (BCT 2.3), ‘Social support (practical)’ (BCT 3.2), ‘Social support (emotional)’ (BCT 3.3), ‘Information about health consequences’ (BCT 5.1), ‘Monitoring of emotional consequences’ (BCT 5.4), ‘Information about emotional consequences’ (BCT 5.6), ‘Prompts/cues’ (BCT 7.1), ‘Habit reversal’ (BCT 8.4), ‘Graded tasks’ (BCT 8.7), ‘Pros and cons’ (BCT 9.2), ‘Comparative imagining of future outcomes’ (BCT 9.3), ‘Social reward’ (BCT 10.4), ‘Self‐reward’ (BCT 10.9), ‘Reduce negative emotions’ (BCT 11.2), ‘Distraction’ (BCT 12.4), ‘Adding objects to the environment’ (BCT 12.5), ‘Body changes’ (BCT 12.6), ‘Verbal persuasion about capability’ (BCT 15.1) and ‘Focus on past success’ (BCT 15.3).

The reasons for exclusion of the six remaining BCTs are outlined in Table 2.

**Table 2:** Short‐listed behaviour change techniques excluded by the codesign team.

Behaviour change technique (BCT)	Reason for exclusion
Goal setting (outcome) (BCT 1.3)	Codesign team members recommended that goals relating to discontinuing BZRA use through a process of gradual dosage reduction over time focus on the behaviour (BCT 1.1) as opposed to the outcome to avoid setting rigid timelines for dosage reduction or a goal of complete discontinuation from the outset.
Action planning (BCT 1.4)	Codesign team members recommended using graded tasks (BCT 8.7) instead of action planning to avoid an inflexible or unachievable approach to gradual dosage reduction.
Commitment (BCT 1.9)	Codesign team members recommended avoiding this BCT as it could be misconstrued that individuals who are not successful with BZRA discontinuation lack commitment.
Salience of consequences (BCT 5.2)	Codesign team members recommended using information about the health consequences (BCT 5.1) and emotional consequences (BCT 5.6) of BZRA discontinuation as opposed to salience of consequences as there was no meaningful or appropriate way of going beyond informing individuals about such consequences in relation to BZRA discontinuation.
Social comparison (BCT 6.2)	Codesign team members recommended avoiding this type of comparison as individual circumstances and experiences of discontinuation and associated withdrawal symptoms are unique and not directly comparable.
Avoidance/reducing exposure to cues for the behaviour (BCT 12.3)	Codesign team members recommended avoiding this BCT as the target behaviour was to discontinue BZRA use.

Abbreviation: BZRA, benzodiazepine receptor agonist.

### 3.2 Developing draft intervention(s) using selected BCTs (Step 5)

At the second codesign team meeting, one additional BCT (‘Credible source’—BCT 9.1) was presented and discussed. This BCT had not been mapped to the TDF in previous work, but was considered relevant to the study based on discussion points raised by the team at the first meeting. Codesign team members agreed to include ‘Credible source’ (BCT 9.1), with team members noting the importance of credible guidance sources in supporting BZRA discontinuation. This resulted in 24 BCTs being selected for inclusion in the intervention (Table 3).

**Table 3:** Recommendations for clinicians in operationalizing behaviour change techniques within the SAFEGUARDING‐BZRAs toolkit.

Theoretical domain	Behaviour change technique (BCT)	Toolkit recommendations for clinicians in operationalizing BCTs
Knowledge	Information about health consequences (BCT 5.1)	Outline the reasons why long‐term BZRA use is not recommended (e.g., dependence, withdrawal and tolerance). Explain the terms dependence, withdrawal symptoms and tolerance in language that patients can understand. Explain the health risks associated with long‐term BZRA use and tailor information according to the patient's age profile (e.g., cognitive impairment and falls/fractures in older adults). Outline the potential for unpredictable adverse effects (e.g., paradoxical reactions) associated with BZRA use, as well as common adverse effects (e.g., drowsiness). Discuss the benefits and risks associated with discontinuing BZRA use.
	Feedback on behaviour (BCT 2.2)	Monitor and provide feedback on the patient's progress with a dosage reduction schedule and adjust if necessary (e.g., encourage the patient to remain on a particular dose if he or she is struggling with withdrawal symptoms).
	Distraction (BCT 12.4)	Encourage patients to identify distraction techniques that they could use as an alternative focus for attention to avoid triggers for BZRA use (e.g., breathing techniques, meditation, exercise, group activities). Encourage patients to try to shift their focus away from the tapering process to normal day‐to‐day activities (i.e., try and work the tapering process into daily life as opposed to living life around the tapering schedule).
Skills	Graded tasks (BCT 8.7)	Set agreed targets for gradually reducing BZRA use with the patient and ensure that any targets are achievable, monitored regularly and adjusted if necessary. Dosage reduction is not always a linear process and maintaining a lower dose for a period of time may be necessary before proceeding with any further dosage reduction. Emphasize the importance of gradual dosage reduction in small increments.
	Habit reversal (BCT 8.4)	Encourage patients to use alternative strategies to manage anxiety and/or insomnia (e.g., exercise, yoga, mindfulness, meditation) in place of BZRAs (unwanted habitual behaviour). Avoid any connotation between this BCT and drug addiction or abuse.
	Body changes (BCT 12.6)	Promote methods of relaxation training to facilitate a gradual dosage reduction process (e.g., yoga, exercise, mindfulness, meditation).
Social/professional role and identity	Social support (emotional) (BCT 3.3^a^) [also included under the ‘social influence’ domain]	Advise on, arrange or provide emotional support to patients undergoing the BZRA dosage reduction process (e.g., dealing with withdrawal symptoms). Encourage patients to identify and develop their own network of social support (e.g., through family, friends, peer support groups) to assist them throughout the BZRA dosage reduction process.
	Social support (practical) (BCT 3.2^a^) [also included under the ‘social influence’ domain]	Advise on, arrange or provide help to patients with the practical aspects of managing BZRA dosage reduction process (e.g., implementing tapering schedule, splitting tablets). Encourage patients to identify and develop their own network of social support (e.g., through family, friends, peer support groups) to assist them throughout the BZRA dosage reduction process.
Beliefs about capabilities	Verbal persuasion about capability (BCT 15.1^a^) [also included under the ‘optimism’ domain]	Tell the person that they can successfully reduce and/or discontinue BZRA use, and help them to overcome any self‐doubts. Tailor verbal persuasion to the individual and their own unique circumstances, and remind them to taper at a rate that they are comfortable with (i.e., avoid overly rapid dosage reduction).
	Focus on past successes (BCT 15.3)	Advise the patient to think about any previous successes in reducing BZRA use. Encourage the patient to think about other previous life challenges that they have overcome (e.g., smoking cessation).
Optimism	Verbal persuasion about capability (BCT 15.1^a^) [also included under the ‘beliefs about capabilities’ domain]	Tell the person that he or she can successfully reduce and/or discontinue BZRA use, and help him or her to overcome any self‐doubts. Tailor verbal persuasion to the individual and his or her own unique circumstances, and remind him or her to taper at a rate that they are comfortable with (i.e., avoid overly rapid dosage reduction).
Beliefs about consequences	Comparative imagining of future outcomes (BCT 9.3)	Advise patients to imagine and compare future outcomes based on discontinuing BZRA use (changed behaviour) versus continuing BZRA use (unchanged behaviour).
	Pros and cons (BCT 9.2)	Advise patients to identify and compare their own individual reasons for wanting (pros) and not wanting to (cons) to discontinue BZRA use.
Reinforcement	Social reward (BCT 10.4)	Provide praise and encouragement if the patient has shown progress with gradual dosage reduction.
	Self‐reward (BCT 10.9)	Encourage self‐praise or self‐reward if the patient has shown progress with gradual dosage reduction. Advise the patient to identify meaningful self‐rewards to mark particular milestones during the dosage reduction process and use them to reward continued progress.
Intentions	No BCTs were included that mapped to intentions.	No BCTs were included that mapped to intentions.
Goals	Goal setting (behaviour) (BCT 1.1)	Set the goal of discontinuing BZRA use through a process of gradual dosage reduction over time. It is important to be realistic, flexible and supportive throughout this process, particularly in cases where patients experience severe or protracted withdrawal symptoms and need to either ‘up dose’ or reinstate the medication for a period of time. Avoid setting rigid timelines for dosage reduction or a goal of complete discontinuation from the outset.
	Review outcome goal(s) (BCT 1.7)	Review the outcome goal in terms of the agreed targets for gradually reducing BZRA use (i.e., graded tasks) with the patient and consider modifying the dosage reduction strategy if necessary. Avoid setting an overly rigid outcome goal of complete BZRA discontinuation from the outset so as to promote a sustained effort towards safe and gradual reduction between the patient and the clinician at a pace that the patient is comfortable with.
	Review behaviour goal(s) (BCT 1.5)	Review the behaviour goal (i.e., discontinuing BZRA use through a process of gradual dosage reduction over time) with the patient and consider modifying the dosage reduction strategy if necessary. Review the use of any alternative behaviours/strategies to manage anxiety and/or insomnia (e.g., exercise, yoga, mindfulness, meditation) in place of BZRAs.
Memory, attention and decision processes	Prompts and cues (BCT 7.1^a^) [also included under the ‘environmental context and resources’ domain]	Encourage the patient to use a reminder (electronic or written) to prompt future dosage reductions and keep track of the overall progress with a dosage reduction schedule.
	Self‐monitoring of behaviour (BCT 2.3^a^) [also included under the ‘behavioural regulation’ domain]	Work with the patient to identify a suitable method (e.g., written, electronic) for them to monitor and record their progress with the dosage reduction strategy.
Environmental context and resources	Adding objects to the environment (BCT 12.5)	Provide evidence‐based educational and support materials on BZRA discontinuation (e.g., tapering schedule). Encourage patients to use a journal to track their progress with the taper. Encourage patients to use stickers or post‐it notes in their home environment as a source of positive affirmations (e.g. ‘your body is healing’). Signpost patients to other relevant and credible resources to facilitate discontinuation process.
	Prompts and cues (BCT 7.1^a^) [also included under the ‘memory, attention and decision processes’ domain]	Encourage the patient to use a reminder (electronic or written) to prompt future dosage reductions and keep track of the overall progress with a dosage reduction schedule.
Social influences	Social support (emotional) (BCT 3.3^a^) [also included under the ‘social/professional role and identity’ domain]	Advise on, arrange or provide emotional support to patients undergoing the BZRA dosage reduction process (e.g., dealing with withdrawal symptoms). Encourage patients to identify and develop their own network of social support (e.g., through family, friends, peer support groups) to assist them throughout the BZRA dosage reduction process.
	Social support (practical) (BCT 3.2^a^) [also included under the ‘social/professional role and identity’ domain]	Advise on, arrange or provide help to patients with the practical aspects of managing the BZRA dosage reduction process (e.g., implementing tapering schedule, splitting tablets). Encourage patients to identify and develop their own network of social support (e.g., through family, friends, peer support groups) to assist them throughout the BZRA dosage reduction process.
Emotion	Reduce negative emotions (BCT 11.2)	Advise on ways of reducing and avoiding stress and other negative emotions to facilitate the dosage reduction process. Encourage patients to avoid negatively positioned accounts and commentaries (e.g., social media) on BZRA discontinuation and withdrawal.
	Information about emotional consequences (BCT 5.6)	Provide information on the positive emotional consequences of discontinuing BZRA use (e.g., reduced anxiety). Provide information on the potential for negative emotional consequences of discontinuing BZRA use, particularly during the initial stages of the dosage reduction process (e.g., increased anxiety, irritability).
	Monitoring emotional consequences (BCT 5.4)	Encourage patients to assess and record their feelings during the dosage reduction process, particularly in terms of the challenges encountered and the associated emotional impact.
Behavioural regulation	Self‐monitoring of behaviour (BCT 2.3^a^) [also included under the ‘memory, attention and decision processes’ domain]	Work with patients to identify a suitable method (e.g., written, electronic) for them to monitor and record their progress with the dosage reduction strategy.
Not applicable	Credible source (BCT 9.1)	Use a credible source to present verbal communication in favour of BZRA discontinuation. A credible source may include an organization, a clinician, an evidence‐based resource or people with lived experience of discontinuing BZRA use.
	[BCT has not been mapped to a theoretical domain]	

Abbreviation: BZRA, benzodiazepine receptor agonist.

^a^ BCT mapped to more than one theoretical domain in the original mapping matrices.

Possible ways in which each of the 24 included BCTs could be operationalized as part of an intervention to support discontinuation of long‐term BZRA use were discussed. Given the number of BCTs, it was suggested that they be presented as part of a toolkit. A toolkit is defined as the packaging of multiple resources that codify explicit knowledge, guidelines, summaries and algorithms whose purpose is to share knowledge, educate and facilitate behaviour change.^30^ This suggestion was accepted by all team members as the most suitable method for presenting the BCTs and enabling BCTs to be selected as part of an individually tailored approach for supporting discontinuation of long‐term BZRA use. The codesign team generated recommendations targeted at primary care‐based clinicians for operationalizing each BCT to support BZRA discontinuation among willing individuals as part of the SAFEGUARDING‐BZRAs toolkit (Supporting sAFE and GradUAl ReDuctIon of loNG‐term BenZodiazepine Receptor Agonist uSe) toolkit (Table 3).

### 3.3 Selecting draft intervention(s) for further testing (Step 6)

As a single intervention was developed comprising a toolkit of BCTs that could be selected as part of an individually tailored approach for supporting discontinuation of long‐term BZRA use among willing individuals, the APEASE criteria were not applied.

## 4 DISCUSSION

This paper describes the development of the SAFEGUARDING‐BZRAs toolkit, which aims to support the safe discontinuation of long‐term BZRA use through gradual dosage reduction among willing individuals. The toolkit was developed to address the lack of appropriately described, theory‐based interventions targeting long‐term BZRA use in primary care.^12^, ^31^ The toolkit is the culmination of a multiphase research project that aligned with the UK Medical Research Council's complex intervention framework^15^ and aimed to systematically develop a theory‐based intervention to support discontinuation of long‐term BZRA use. In addition to incorporating evidence and theory through the preceding systematic review^12^ and TDF‐based qualitative interviews with individuals with experience (current or previous) of long‐term BZRA use,^16^ the development process used a codesign approach.^25^ This involved an active partnership between experts by experience, researchers and clinicians, and has helped to ensure that the toolkit's development was informed by a combination of both lived and clinical experience relating to BZRA discontinuation.

The SAFEGUARDING‐BZRAs toolkit comprises 24 BCTs from the BCT taxonomy (v1).^21^ The comprehensive nature of the toolkit and its inclusion of multiple BCTs are reflective of the challenging nature of BZRA discontinuation as a target behaviour. This is attributable to individuals' unique experiences of BZRA use (e.g., reasons for initiating and maintaining BZRA use) and the range of barriers that can impact on individuals attempting discontinuation of long‐term BZRA use.^16^ This is consistent with previous research on mental health service users' experiences of discontinuing psychotropic medication.^32^ The collection of BCTs within the toolkit offers a library of options for individuals and clinicians to use as part of an active partnership in developing a gradual dosage reduction strategy that should include regular monitoring and support. The retrospective coding of interventions in our previous systematic review^12^ identified a comparatively smaller repertoire of BCTs, whereby the number of BCTs per intervention ranged between 4 and 8 BCTs and 17 unique BCTs were identified across included studies. This may have partly been attributable to the brief intervention format, as well as the lack of detailed reporting within included studies and the absence of any formal taxonomy for describing the interventions' content. However, a number of previously identified BCTs were questioned by the codesign team in terms of suitability for individuals attempting BZRA discontinuation. For example, one previous study included a peer champion story (coded as ‘Social comparison’—BCT 6.2) to encourage participants to attempt BZRA discontinuation.^33^ Granted this was framed as a positive success story and the intervention has shown positive effects,^33^ however, codesign team members expressed the need for caution in individuals comparing their situation and any progress (or lack thereof) with BZRA discontinuation to others as experiences of withdrawal symptoms can vary substantially. Team members also cautioned against planning for BZRA discontinuation as the outcome from the outset is based on individual members' lived experience of BZRA discontinuation and the wider network of BZRA users that they have engaged with through peer support‐related activities; the discontinuation process is often not linear. Therefore, it was recommended that goal setting focus on BZRA discontinuation as a behaviour (BCT 1.1) as opposed to an outcome (BCT 1.3) and this should use graded tasks (BCT 8.7) as opposed to action planning (BCT 1.4).

In addition to providing a comprehensive range of BCTs to support safe and gradual reduction of BZRA use, the toolkit's component BCTs are linked to determinants of behaviour change from the TDF.^27^ This could help in tailoring the selection of BCTs to key theoretical domains that are perceived to act as barriers to BZRA discontinuation at an individual level. For example, where patients lack appropriate support to manage the practical aspects of the BZRA dosage reduction process, such as implementing tapering schedules or splitting tablets, (i.e., barrier under the ‘Social influences’ domain), this could be addressed through ‘Social support (practical)’ (BCT 3.2). The linking of BCTs to theoretical determinants of behaviour change represents a particularly novel aspect of the SAFEGUARDING‐BZRAs toolkit as our previous systematic review highlighted that several domains were either not evident (e.g., ‘emotion’) or seldom evident (e.g., ‘optimism’, ‘beliefs about capabilities’) in interventions targeting long‐term BZRA use.^12^ This has implications in terms of the interventions' potential to target relevant determinants of behaviour change, such as negative perceptions and emotions towards BZRA discontinuation, that have previously been reported among individuals taking these medications on a long‐term basis.^34^

‘Intentions’ is the only theoretical domain not explicitly targeted by BCTs within the toolkit. This is partly attributable to the two BCTs (‘Behavioural contract’ [BCT 1.8], ‘Commitment’ [BCT 1.9]) that were originally mapped to this domain in previous research.^27^ For example, codesign team members recommended avoiding ‘Commitment’ (BCT 1.9) as a BCT as it could be misconstrued that individuals who are not successful with BZRA discontinuation lack commitment. However, it is important to remember that the original mapping matrices were not developed with BZRA discontinuation as the target behaviour.^20^, ^27^ It is therefore possible that other BCTs within the toolkit (e.g., social support), or a synergistic effect between BCTs, may help to target this domain indirectly. The lack of direct targeting of ‘intentions’ is also reflective of the fact that the SAFEGUARDING‐BZRAs toolkit is intended to be used as part of an active partnership between individuals who themselves want to discontinue long‐term BZRA use and their clinicians. The authors do not encourage, or support, forced withdrawal or discontinuation of long‐term BZRA use. The toolkit may provide a useful catalyst for collaborative, person‐centred approaches to treatment decisions between individuals and their clinicians and help to prevent situations whereby individuals who have decided that they no longer want to continue taking psychotropic medication encounter challenges in accessing professional support and having their autonomy and choice regarding treatment options respected.^35^

Future work will look to develop an accompanying tool to help individuals and their clinicians identify priority domains that need to be targeted in designing a tailored BZRA discontinuation plan at an individual level. For example, the research team is currently in the process of developing and validating a TDF‐based questionnaire that aims to examine mediators of behaviour change relating to discontinuing long‐term BZRA use. We also intend to examine the potential role of smartphone technology in providing an interactive and tailored mode of delivery. This would serve to advance the current evidence base as many previous interventions have relied on noninteractive, paper‐based resources (e.g., information booklets) with limited potential for tailoring according to individual patient needs.^8^, ^12^, ^31^ This will ultimately help in selecting the most relevant BCTs to support an individual in attempting BZRA discontinuation.

As more work is undertaken and the cumulative evidence base for individual BCTs in changing specific behaviours continues to expand, the list of included BCTs may need to be updated accordingly. For example, there is currently a lack of evidence to support pharmacological interventions (e.g., antidepressants, anticonvulsants) to facilitate discontinuation of long‐term BZRA use.^36^ However, if effective treatments are identified, then ‘pharmacological support’ (BCT 11.1) will need to be added to the toolkit. Experience with the toolkit in clinical practice should help to refine the toolkit and identify which BCTs are most likely to be effective in different clinical scenarios. Therefore, the toolkit is intended to be a living resource and the authors welcome feedback based on user experience. We plan to review and update the toolkit at regular intervals.

### 4.1 Strengths and limitations

The main strength of this study is that the development of the SAFEGUARDING‐BZRAs toolkit followed a systematic approach that was underpinned by evidence and theory in accordance with the Medical Research Council's complex intervention framework.^15^ The detailed reporting of the toolkit's development and components, as well as the inclusion of an underpinning theoretical framework of behaviour change, has been largely absent in previous BZRA‐related research.^12^, ^31^ For example, in our previous systematic review of brief interventions targeting long‐term BZRA use, only one of the included studies described the intervention development process and the techniques used to achieve behaviour change.^33^, ^37^ However, a formal taxonomy was not used to describe the intervention's content, which limits the potential for accurate replication. The use of BCTs from the BCTTv1^21^ in specifying the content of the SAFEGUARDING‐BZRAs toolkit may help in addressing this and facilitating its uptake and implementation by service users, clinicians and researchers. The use of a codesign approach involving an active partnership between experts by experience, researchers and clinicians has helped to ensure that the toolkit's development was grounded in relevant clinical and lived experience. In terms of limitations, it is possible that a different group of individuals may have developed a different type of intervention. We attempted to overcome this by using a structured and transparent approach to decision‐making (e.g., using a priori decision rules) during the codesign team meetings. It must be noted that there is insufficient evidence to recommend that a particular approach or set of actions is essential to produce an effective intervention.^38^ Although the current iteration of the toolkit is targeted at primary care‐based clinicians working with community‐based individuals, it could be adapted for use by other relevant groups including psychiatrists and peer support workers. Further work is needed to evaluate how the toolkit can be implemented in practice. The use of mobile health solutions (e.g., smartphone applications) as a mode of intervention delivery should be considered as these forms of technology can increase access to self‐management strategies while also enhancing mental health care by delivering real‐time assessments and personalized feedback.^39^ However, any such solutions would need to be assessed in terms of usability and acceptability by service users and clinicians.

## 5 CONCLUSION

This paper reports on the development of the SAFEGUARDING‐BZRAs toolkit using a codesign approach. The toolkit addresses identified limitations of previous research (e.g., lack of detailed intervention description, lack of appropriate theoretical underpinning) and adds to the body of literature relating to behaviour change interventions targeting discontinuation of long‐term BZRA use. Further work is needed to examine the use of smartphone technology in delivering the toolkit in practice, its usability and acceptability by service users and clinicians and its potential to effectively support safe and gradual reduction of long‐term BZRA use among willing individuals.

## AUTHOR CONTRIBUTIONS

*Conceptualization*: Tom Lynch, Cristín Ryan, Cathal Cadogan. *Methodology*: Tom Lynch, Cristín Ryan, Cathal Cadogan. *Formal analysis and investigation*: Tom Lynch, Cristín Ryan, Colin Bradley, D. Foster, Christy Huff, Sharon Hutchinson, Nicole Lamberson, Lily Lynch, Cathal Cadogan. *Writing–original draft preparation*: Tom Lynch, Cristín Ryan, Cathal Cadogan. *Writing–review and editing*: Tom Lynch, Cristín Ryan, Colin Bradley, D. Foster, Christy Huff, Sharon Hutchinson, Nicole Lamberson, Lily Lynch, Cathal Cadogan. *Funding acquisition*: Tom Lynch, Cristín Ryan, Cathal Cadogan. *Resources*: Tom Lynch, Cristín Ryan, Cathal Cadogan. *Supervision*: Cristín Ryan, Cathal Cadogan. All authors read and approved the final manuscript.

## CONFLICT OF INTEREST

The authors declare no conflict of interest.

## Supporting information

Supplementary information.

## Data Availability

Research data are not shared due to privacy or ethical restrictions.
