# Supplementary material for: Supporting safe and gradual reduction of long‐term benzodiazepine receptor agonist use: Development of the SAFEGUARDING‐BZRAs toolkit using a codesign approach
Source: Health Expect. 2022 Jun 7;25(4):1904–18. doi: 10.1111/hex.13547 (PMC9327818; doi:10.1111/hex.13547)
Supplement: Supplementary file 1 — Supplementary information. [file HEX-25--s001.docx]

Appendix S1 Behaviour change techniques that were reviewed in developing the initial long-list for presentation at the co-design team meetings

| **Theoretical domain** | **Behaviour change technique** | **Include/exclude** | **Reason for exclusion** |
| --- | --- | --- | --- |
| Knowledge | 5.1 Information about health consequences | Include | N/A |
|  | 2.6 Biofeedback | Exclude | No known external monitoring device for biofeedback in a BZRA context |
|  | 12.1 Restructuring the physical environment | Exclude | Beyond the scope of the project/ intervention |
|  | 12.2 Restructuring the social environment | Exclude | Beyond the scope of the project/intervention |
|  | 12.3 Avoidance/reducing exposure to cues for the behaviour | Include | N/A |
|  | 12.4 Distraction | Include | N/A |
|  | 12.5 Adding objects to the environment | Include | N/A |
|  | 12.6 Body changes | Include | N/A |
|  | 2.2 Feedback on behaviour | Include | N/A |
| Skills | 8.7 Graded tasks | Include | N/A |
|  | 8.1 Behavioural practice/ rehearsal | Exclude | Not applicable to tapering BZRA use |
|  | 8.4 Habit reversal | Include | N/A |
|  | 12.6 Body changes | Include | N/A |
|  | 8.3 Habit formation | Exclude | N/A |
| Social/Professional role and identity | 3.1 Social support (unspecified) | Exclude | Other more specific forms of social support included instead (practical, emotional) |
|  | 3.2 Social support (practical) | Include | N/A |
|  | 3.3 Social support (emotional) | Include | N/A |
|  | 15.1 Verbal persuasion about capability | Include | N/A |
| Beliefs about capabilities | 15.1 Verbal persuasion about capability | Include | N/A |
|  | 15.3 Focus on past success | Include | N/A |
| Optimism | 15.1 Verbal persuasion about capability | Include | N/A |
| Beliefs about consequences | 5.6 Information about emotional consequences | Include | N/A |
|  | 5.2 Salience of consequences | Include | N/A |
|  | 16.1 Imaginary punishment | Exclude | Not applicable to tapering BZRA use |
|  | 5.5 Anticipated regret | Exclude | Not applicable to tapering BZRA use |
|  | 5.3 Information about social and environmental consequences | Exclude | Not applicable to tapering BZRA use |
|  | 9.3 Comparative imagining of future outcomes | Include | N/A |
|  | 10.11 Future punishment | Exclude | Not applicable to tapering BZRA use |
|  | 9.2 Pros and cons | Include | N/A |
|  | 16.2 Imaginary reward | Exclude | Not applicable to tapering BZRA use |
| Reinforcement | 10.11 Future punishment | Exclude | Not applicable to tapering BZRA use |
|  | 10.9 Self reward | Include | N/A |
|  | 14.8 Reward alternative behaviour | Exclude | Beyond the scope of the project/intervention |
|  | 10.8 Incentive (outcome) | Exclude | Beyond the scope of the project/intervention |
|  | 14.4 Reward approximation | Exclude | Beyond the scope of the project/intervention |
|  | 14.6 Situation specific reward | Exclude | Beyond the scope of the project/intervention |
|  | 10.2 Material reward (behaviour) | Exclude | Beyond the scope of the project/intervention |
|  | 10.4 Social reward | Include | N/A |
|  | 10.3 Non-specific reward | Exclude | Beyond the scope of the project/intervention |
|  | 14.1 Behaviour cost | Exclude | Beyond the scope of the project/intervention |
|  | 14.10 Remove punishment | Exclude | Not applicable to tapering BZRA use |
|  | 14.2 Punishment | Exclude | Beyond the scope of the project/intervention |
|  | 14.3 Remove reward | Exclude | Beyond the scope of the project/intervention |
|  | 7.8 Associative learning | Exclude | Beyond the scope of the project/intervention |
| Intentions | 1.9 Commitment | Include | N/A |
|  | 1.8 Behavioural contract | Exclude | Beyond the scope of the project/intervention |
| Goals | 1.1 Goal setting (behaviour) | Include | N/A |
|  | 1.3 Goal setting (outcome) | Include | N/A |
|  | 1.7 Review outcome goal(s) | include | N/A |
|  | 1.5 Review behaviour goal(s) | Include | N/A |
|  | 1.4 Action planning | Include | N/A |
| Memory, attention and decision processes | 2.3 Self-monitoring of behaviour | Include | N/A |
|  | 2.4 Self-monitoring of outcome(s) of behaviour | Exclude | Focused on the target behaviour as opposed to the outcome of that behaviour given the potential for withdrawal symptoms during initial reduction/discontinuation |
|  | 2.5 Monitoring outcome(s) of behaviour by others without feedback | Exclude | Beyond the scope of the project/intervention |
|  | 1.4 Action planning | Include | As above |
|  | 7.1 Prompts/cues | Include | As above |
|  | 7.3 Reduce prompts/cues | Exclude | Beyond the scope of the project/intervention |
| Environmental, context and resources | 12.1 Restructuring the physical environment | Exclude | Beyond the scope of the project/intervention |
|  | 7.2 Cue signalling reward | Exclude | Beyond the scope of the project/intervention |
|  | 7.1 Prompts/cues | Include | N/A |
|  | 12.2 Restructuring the social environment | Exclude | Beyond the scope of the project/intervention |
|  | 12.3 Avoidance/reducing exposure to cues for the behaviour | Include | N/A |
| Social influences | 6.2 Social comparison | Include | N/A |
|  | 6.3 Information about others approval | Exclude | Potential to stigmatise medication use |
|  | 3.3 Social support (emotional) | Include | N/A |
|  | 3.2 Social support (practical) | Include | N/A |
|  | 16.3 Vicarious consequences | Exclude | Potential to stigmatise medication use |
|  | 12.2 Restructuring the social environment | Exclude | Beyond the scope of the project/intervention |
|  | 6.1 Demonstration of the behaviour | Exclude | Not applicable to tapering BZRA |
|  | 13.1 Identification of self as a role model | Exclude | Not applicable to tapering BZRA |
|  | 10.4 Social Reward | Include | N/A |
| Emotions | 11.2 Reduce negative emotions | Include | N/A |
|  | 5.6 Information about emotional consequences | Include | N/A |
|  | 5.4 Monitoring of emotional consequences | Include | N/A |
|  | 3.3 Social support (emotional) | Include | N/A |
| Behavioural regulation | 2.3 Self-monitoring of behaviour | Include | N/A |
